# Implications of COVID-19 for resumption of sport in South Africa: A South African Sports Medicine Association (SASMA) position statement – Part 2

**DOI:** 10.17159/2078-516X/2020/v32i1a8986

**Published:** 2020-01-01

**Authors:** DA Ramagole, DC Janse van Rensburg, L Pillay, P Viviers, P Zondi, J Patricios

**Affiliations:** 1 Section Sports Medicine & Sport, Exercise Medicine and Lifestyle Institute (SEMLI), Faculty of Health Sciences, University of Pretoria, Pretoria, South Africa; 2 Medical Board Member, International Netball Federation, Manchester, UK; 3 Campus Health Service, Stellenbosch University, South Africa; 4 Institute of Sport and Exercise Medicine, Division of Orthopaedic Surgery, Department of Surgical Sciences, Faculty of Medicine and Health Sciences, Stellenbosch University, South Africa; 5 FIFA Medical Centre of Excellence, South Africa; 6 Sports Science Institute of South Africa, Newlands, South Africa; 7 South African Sports Confederation and Olympic Committee, Medical Advisory Committee; 8 Wits Sport and Health (WiSH), Faculty of Health Sciences, University of the Witwatersrand, Johannesburg, South Africa

**Keywords:** athlete, prolonged positive test, manifestation

## Abstract

The lockdown regulations due to the coronavirus pandemic (COVID-19) that broke out towards the end of 2019 and continued to spread throughout most countries in the world had a negative effect on the sporting community. The South African government eased the lockdown rules to Level 1 from 21 September 2020. In Part 2 of this Position Statement of the South African Sports Medicine Association (SASMA), the authors address the position regarding the safe return to sports for athletes who were infected by the virus. An update on clinical manifestations and multi-organ involvement, testing for Severe Acute Respiratory Syndrome Coronavirus 2 (SARS-CoV-2), prolonged positive real time polymerase chain reaction (RT-PCR) and the role of quantitative real time polymerase chain reaction (RT-qPCR) in informing return to sports, grading of disease severity, individualised management of infected athletes and graduated return to play guidelines (GRTP) is provided. The authors also share thoughts on athletes with disabilities, immunisation, the using of masks during exercise and utilising biologically safe environments (BSE). Finally the SASMA Guidelines for Safe RTP in Level 1 lockdown during SARS-Cov-2 are introduced.

The intense scientific focus and medical scrutiny on the Severe Acute Respiratory Syndrome Coronavirus 2 (SARS-CoV-2) pathogen and COVID-19 disease have resulted in an exponential increase in evidence-based publications, case studies and reviews.^[1,2]^ This has necessitated regular reappraisal of scientific evidence and best practice recommendations. This paper represents the first reassessment by SASMA, and further updates will be forthcoming as new evidence emerges.

The lockdown rules were relaxed to Level 2 from midnight on 17 August 2020, allowing for more movement and training in small groups without spectators, but with concomitant stringent measures to reduce the spread of SARS-CoV-2 infections. These measures included regular hand washing, respiratory etiquette, the wearing of masks, and the maintenance of physical (social) distancing.

On 16 September 2020, President Cyril Ramaphosa announced that South Africa would further ease restrictions and enter into Level 1 lockdown. At the time of the submission of this update, the Level 1 regulations and their impact on sport had not yet been gazetted.

The purpose of this document is to highlight areas in which there have been developments that may influence the management of athletes returning to sport amidst the ongoing COVID-19 pandemic. It is not a detailed clinical review of COVID-19 and should be used as supplementary material to inform decision-making. In this document updates in the following clinical areas are provided:

- Clinical manifestations and multi-organ involvement
- Testing for SARS-CoV-2
- Prolonged positive real-time polymerase chain reaction (RT-PCR) and the role of quantitative real-time polymerase chain reaction (RT-qPCR) in informing return to sports (RTP)
- Grading of disease severity, individualised management of infected athletes and graduated return to play guidelines (GRTP)
- Other considerations:
  ○ Athletes with disabilities
  ○ Immunisation
  ○ The use of masks during exercise
  ○ Biologically Safe Environments (BSE)
- SASMA Guidelines for safe RTP in Level 1 lockdown during SARS-CoV-2

## Clinical manifestations of illness

**Fig. 1:**
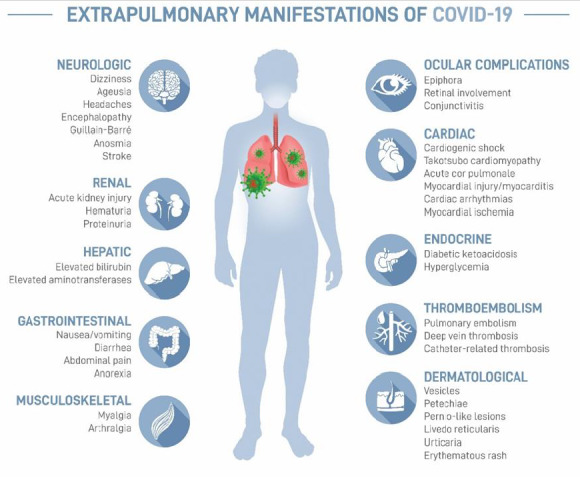
Extra-pulmonary manifestations of COVID-19. (Infographic designed by University of Pretoria, Creative Studios, Marizanne Booyens © 2020)

COVID-19 is known for the initial pulmonary manifestations, but research has shown extensive extra-pulmonary involvement including cardiac abnormalities, hepatocellular damage, ocular problems, dermatological manifestations, renal complications, and neurologic problems, amongst others. Common extra-pulmonary manifestations of COVID-19 include ophthalmic complications, such as conjunctivitis, conjunctival congestion and retinal involvement in some cases.^[3]^ There are also accompanying deficits in both muscle strength and endurance, likely due to the pro-inflammatory effects of the viral infection and the deconditioning that occurs during the convalescent period.^[4]^ The infographic below (Fig. 1) shows the multi-organ manifestation of the illness.

This multi-organ involvement can be detrimental to athletes leading to prolonged illness and a delay in the return to their prior level of performance. Multi-organ involvement may manifest despite a relatively benign initial COVID-19 presentation and be disclosed by means of exertion. Athletes with prolonged illnesses or those who are struggling to progress through a GRTP programme must be investigated for extra-pulmonary complications.

Return to play guidelines should be adapted to accommodate for adequate management of any complications and allow for full recovery before the resumption of vigorous activity.

The impact of the pandemic on individual and societal mental wellbeing has been particularly significant. It has also been noted that this pandemic has affected the livelihood of people including healthcare workers regarding issues on income and uncertainties about job security. This can precipitate depression, anxiety, insomnia and other mental health issues in certain individuals.^[5]^ It is therefore recommended that mental health assessments should be part of the holistic approach in the management of this ailment.

## Testing for SARS-CoV-2

A positive diagnosis is confirmed by the presence of viral RNA detected by RT-PCR testing. Although this test has typically been conducted on secretions obtained from either a nasopharyngeal or oropharyngeal swab, a recent study has shown that detection of the virus in saliva has a similar sensitivity. In countries where saliva tests are available, individuals who suspect infection or exposure can self-administer the test, decreasing the burden on testing laboratories, and decreasing the risk of transmitting illness to healthcare workers administering tests.^[6]^

## Prolonged positive tests in athletes

Part 1 of the SASMA position statement outlines guidelines for monitoring and clinical evaluation, including referral for further cardiology and respiratory investigations.^[7]^ A particular quandary for medical staff relates to prolonged positive tests in athletes who have recovered from illness, are asymptomatic, and are otherwise ready to return to training. A study by Carmo et al. suggests that some patients may present with prolonged positive RT-PCR tests after SARS-CoV-2 infection due to a slower immune response although this has not been associated with severe disease.^[8]^ Prolonged positive tests have also been reported in healthcare workers, and this may be attributed to persistent shedding of the virus.^[9]^

In asymptomatic patients with prolonged positive test results, the RT-qPCR, which confirms the quantity of the virus in a swab, offers the potential to assist in deciding whether patients are still infectious or if the amount of the virus detected is meaningful. This is done by reporting the cycle threshold (Ct) value, which is inversely proportional to the viral load. Emerging research shows that patients with a Ct value above 35 do not excrete infectious viral particles and can therefore be de-isolated.^[10]^

Further evidence suggests that an increase of approximately 3.3 in Ct value equates to a tenfold decrease in the viral load. Used in conjunction with other investigations and a clinical assessment, the Ct value may be a helpful tool to inform decisions on RTP in athletes with prolonged positive tests. Immunoglobulins detected in serological analysis may be another clinically useful indicator. The presence of immunoglobulin M and G (IgM and IgG) antibodies can be used to monitor the progression of the disease. IgM antibodies are produced early in the infection stage and decline with time, while IgG antibodies persist for up to 16 months in previously known SARS infections.^[1]^

The presence of IgG and low or absent IgM can therefore be used as an indicator of past inactive infection.^[11]^

## Grading of illness severity

The Canadian Olympic and Paralympic Working Committee has published clinical guidelines grading COVID-19 into three levels of infection that need to be managed differently:^[12]^

1. *COVID-19 positive and asymptomatic*. These athletes have mild disease and symptom resolution within 10 days.
2. *COVID-19 positive and prolonged course*. These athletes have regional or systemic symptoms lasting more than 10 days, or severe symptoms that may require hospitalisation.
3. *COVID-19 positive with symptoms developing during Graduated Return to Play Progression (GRTPP)*.

According to these guidelines, individualised management of affected athletes is important and can be stratified as follows:

1. *COVID-19 positive and asymptomatic or mild localised disease*
  - Self-isolation
  - Rest and recovery (no exercise)
  - De-isolation after 10 days
  - A careful history and clinical evaluation by a physician
  - Based on clinical assessment, and in the presence of pre-existing comorbidities, consider blood Troponin levels and 12-lead resting electrocardiograph (ECG), inflammatory markers, e.g. C-reactive protein (CRP), full blood count (FBC), Troponin, renal assessment and spirometry
  - In the absence of comorbidities, athletes may begin a GRTPP if they are symptom-free and have not been on any medication for seven days
2. *COVID-19 positive with prolonged course*
  - Self-isolation
  - Rest and recovery (no exercise)
  - When asymptomatic, full history and medical assessment are required including:
    ○ FBC, CRP, Troponin, Creatinine
    ○ ECG, Echocardiogram
    ○ Spirometry
  - Athletes with an abnormal cardiac evaluation should be referred to a cardiologist. The assessment may include effort ECG, echo and cardiac magnetic resonance imaging (MRI).
  - Those with an abnormal spirometry should be referred to a pulmonologist.
  - Those with no evidence of cardiac or pulmonary involvement can start the GRTPP after seven days of being symptom-free and as long as they are not on medication for COVID-19-related symptoms.
3. The third group is athletes who were *COVID-19 positive and developed symptoms during the GRTPP*. These athletes may present with the following symptoms during training:
  - Elevated resting heart rate
  - Elevated heart rate at submaximal exertion
  - Increased shortness of breath (SOB) during exertion or increased rating of perceived exertion (RPE)

These athletes should not exercise and continue with rest while symptoms persist. They will need re-evaluation and should be assessed by a sports cardiologist who may refer the athlete for a stress ECG and cardiac MRI to exclude cardiac oedema^[13]^ before resumption of the GRTPP.

**Fig. 2:**
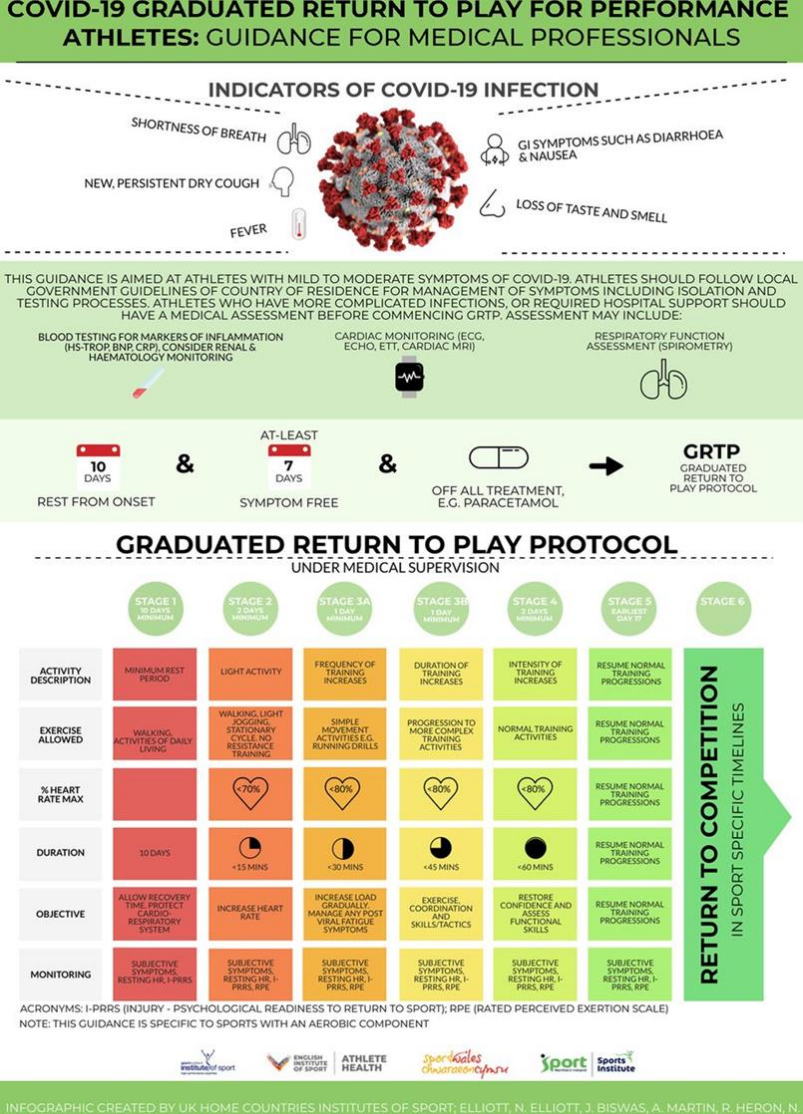
COVID-19 Graduated Return to Play for Performance Athletes: Guidance for Medical Professionals (Permission to reuse granted by BMJ publishing, BJSM, English Institute of Sport and Sport Scotland Institute of Sport; 03 Oct 2020)

Fig. 2 by Elliot et al. ^[14]^ gives an example of a GRTPP introduced after COVID-19 infection. Importantly, the GRTPP must be done under medical supervision.

## Other considerations

### Athletes with disabilities

When planning to resume sport and physical activities, athletes with disabilities should receive extra considerations and precautionary measures. These athletes remain at a higher risk for more severe forms of illness, and considerations for different types of disabilities need to be put in place.^[15]^ Custodians of disability sport will need to customise RTP where clinically relevant.

### Immunisation

Immunisation, as the fastest means of achieving herd immunity, presents the most promising way of returning to sport in a normalised environment. Several vaccines are being developed by a number of pharmaceutical companies globally. Lurie et al. describe the types of vaccines and different approaches being trialled, specifically: live attenuated, inactivated, replicating or non-replicating viruses, with varying use of DNA, RNA or protein subunits to develop the vaccine.^[16]^ Global collaboration towards developing a vaccine against the SARS-CoV-2 virus has enabled rapid progress with clinical phase 3 trials underway in the UK, America, Brazil and South Africa.^[17]^

### Views on the wearing of masks during exercise

The wearing face masks is one of the key strategies to minimise infection. Careful consideration should be given to the type of masks used, factoring in comfort, hygiene, level of exertion and underlying comorbidities.^[18]^ Cloth masks are generally recommended for exercise as they are easier to breathe through and to keep clean by regular washing.^[19]^

SASMA’s position on mask-wearing, particularly where the athletes have not had a recent negative COVID-19 PCR test, is stated below:

- Masks should be worn by all athletes to and from training and matches, as well as in the changeroom
- Where exercise is performed at low to moderate intensities and masks can be tolerated, they should be worn. At high intensities of exercise, where breathing difficulties may occur, athletes may exercise without masks but should attempt to increase physical distancing to a minimum of five m for runners and up to 10 m for cyclists as per a simulation study done by Blocken et al.^[20]^ This study also advises on maintaining physical distancing of 1.5 m apart for people who are stationary, and the distance increased to 5–10 m depending on the level of exertion when exercising with a mask is not possible

### Biologically Safe Environments

The Premier Soccer League (PSL) in South Africa has implemented bringing teams back in a biologically safe environment (BSE) or “bubble”. The BSE was achieved in a phased manner. This could only be done once the government approved the application for resumption of training and matches behind closed doors.

This phased approach is added as a supplementary document: Annexure 1.

### SASMA guidelines for safe RTP in Level 1 lockdown during COVID-19 and SARS-CoV-2

- Thorough pre-participation medical and physical screening
- Follow guidelines on GRTPP for infected athletes
- Persistent positive test – request RT-qPCR. This will indicate Ct values, and values over 35 are considered less meaningful and unlikely to be infectious
- Stringent hand hygiene and avoidance of face touching
- Wearing of masks
- Frequent cleaning of all equipment
- Social distancing at 1.5 m apart for stationary people, and distance increased to 5–10 m depending on the level of exertion without a mask
- Sports with no spectators (as per current government guidelines)
- Limitation of travelling according to government guidelines

## Conclusion

In the absence of herd immunity, COVID-19 remains a public health risk in South Africa and globally. Although encouraged by the declining number of positive tests, everyone should remain vigilant and responsible during the ongoing pandemic. As athletes prepare to return to sport, this must be done cautiously with collaboration between medical and coaching staff. Athletes must be cleared to participate after an infection and be gradually assisted to good health and performance.

SASMA reminds all stakeholders to continue with hand hygiene, respiratory etiquette, physical distancing and importantly, all South Africans are encouraged to keep active and healthy during this period.

## Supplementary Information
