# Supplementary material for: Implications of COVID-19 for resumption of sport in South Africa: A South African Sports Medicine Association (SASMA) position statement – Part 2
Source: S Afr J Sports Med. 2020 Jan 1;32(1):v32i1a8986. doi: 10.17159/2078-516X/2020/v32i1a8986 (PMC9924523; doi:10.17159/2078-516X/2020/v32i1a8986)
Supplement: Supplementary file 1 [file 2078-516X-32-v32i1a8986-s001.pdf]

Implications of COVID-19 for resumption of sport in South Africa: A South African Sports Medicine Association (SASMA) position statement – Part 2

APPENDIX A
Biologically Safe Environment (BSE) approach
Creating a 'Bubble'

Timelines of the phases used to create biologically safe environments were guided by government permissions and directives issued by the professional soccer league. Four phases were identified. Phase 1 to 3 occurred from 15 June 2020 to 3 August 2020 (during Level 3 of the lockdown). Phase 4 (BSE) occurred 4 August 2020 to 21 September 2020 (during Levels 3 and 2 of the lockdown). The following sequence was followed:

Phase 1

Before resumption of training all players and staff had polymerase chain reaction (PCR) for COVID-19 testing done. Athletes with positive test results were isolated according to the guidelines of the National Institute for Communicable Diseases (NICD) and the Department of Health (DoH) while the players with negative test results, and support staff, resumed activities. Strict protocols were put in place: sanitising of the facility, daily questionnaire screening of all staff before coming to training, players arriving at training venues already in their kits or changing outside and not in change rooms (to promote social distancing), regular hand sanitising, each player using their own water bottle, no on-site eating (only take-away meals given), wearing of masks (when not training), and avoiding of in-room team meetings. A compliance and health officer was appointed in each club. Clinical management protocols of positive cases were adjusted as evidence became available or NICD and DoH guidelines had changed. This included clearance which was necessary from a medical doctor and included clinical evaluation, re-evaluation of rest electrocardiograms (ECG), blood investigations, peak flow test and repeat PCR testing.

Phase 2

As training resumed, all team doctors had to do medical examinations on players and staff that were identified as entering the BSE. These medicals included resting ECG and peak flow measurements. This also allowed teams to educate and reinforce measures put in place to mitigate risk (social distancing, hand hygiene, respiratory hygiene, mask-wearing when not training and playing). Essentially, players and staff had to get used to a "new normal".

Phase 3

Before entering the BSE, teams had to fulfil specific criteria: (a) ensure all medicals including ECG and peak flow were done and all persons cleared as healthy to participate in sport or identified as high-risk persons to contract COVID-19 (b) have a COVID-19 test done 48hrs before entering the BSE and while awaiting these results, to be housed in a "mini bubble" to avoid inadvertent exposure to COVID-19. The "mini bubble" was created where all those entering the BSE, would be individually housed overnight in a team camp, hotel, or clubhouse. This created a "buffer zone" before entering the BSE. Only persons testing negative were allowed into the BSE. Those persons who had tested positive had to follow the NICD and DoH guidelines (at that point) of isolation for 14 days and then be cleared by a doctor after clinical evaluation, repeat ECG and blood investigations. Cardiac echo was done if clinically indicated. Athletes with persistent positive test results were evaluated by comparing the cycle threshold values of their initial PCR test and second PCR test and were allowed into the BSE if those values were >34 (suggesting viral shedding and non-infective). Teams were advised on precautions to take during travelling (regular hand sanitising, wearing gloves in the airport and disposing of them afterwards, maintaining social distancing, constant mask-wearing). The same process applied to match officials.

Final Phase (in the BSE): Phase 4

All persons in the BSE were given strict instructions regarding conduct in the BSE. Teams were housed in hotels dedicated to team use and ate in isolated environments. Each hotel had a dedicated COVID-19 doctor (aside from team doctors) to assist in dealing with medical and injury-related problems. Hotel gyms and chlorinated pools were utilised for rehabilitation and recovery only, with strict adherence to bookings, number of persons (social distancing), mask-wearing and cleaning by the hotel staff and team members. In the BSE, daily questionnaire screening continued – those "red flagged" by the screening were isolated and managed accordingly. These persons were managed symptomatically according to their clinical presentation, and this included a second COVID-19 PCR testing. All other persons in the BSE (media, drivers, hotel staff) were allowed entry to the bubble based on a negative PCR COVID-19 test. Once an individual left the bubble, they could not return. Only those that had to leave the bubble for medical-related issues or extreme circumstances were allowed to do so, and each of these requests were individually evaluated. Any movement had to request permission from the league chief medical officer (CMO) and compliance officer for approval. This was also true for any delivery that needed to be received as its contents had to be evaluated and be sanitised by the hotel. Match commissioners also needed to record teams screening results and compliance in the BSE. On match days, social distancing was

practised on the side-lines with substitutes sitting in the stands. The coaching and medical staff were on the side-lines, all (except the head coach) wearing masks. Paramedics wore full personal protective equipment. Any suspicious cases were immediately isolated, and PCR tested to exclude COVID-19. All screening, as per government regulation, needed to be kept by teams for 6 months.

Results of the BSE

The strict compliance measures and clinical surveillance put in place succeeded to identify and manage not only possible COVID-19 related illness, but other illnesses as well without leading to a BSE related epidemic. This can only be achieved if all persons understand that the protocols are in place for “safety first” reasons and if there is genuine compliance to them. This has led to a successful implementation of the BSE protocols.
